# A Scoping Review of The Efficacy of Virtual Reality and Exergaming on Patients of Musculoskeletal System Disorder

**DOI:** 10.3390/jcm8060791

**Published:** 2019-06-04

**Authors:** Hui-Ting Lin, Yen-I Li, Wen-Pin Hu, Chun-Cheng Huang, Yi-Chun Du

**Affiliations:** 1 Department of Physical Therapy, I-Shou University No. 8, Yida Road, Yan-chao District, Kaohsiung 82445, Taiwan; huitinglin@isu.edu.tw (H.-T.L.); ssttaarrtt7616274@gmail.com (Y.-I.L.); 2 Department of Bioinformatics and Medical Engineering, Asia University. 500, Lioufeng Road, Wufeng, Taichung 41354, Taiwan; wenpinhu@asia.edu.tw; 3 Department of Rehabilitation, E-DA Hospital, No.1, Yida Road, Yan-chao District, Kaohsiung 82445, Taiwan; ed109049@edah.org.tw; 4 Department of Electrical Engineering, Southern Taiwan University of Science and Technology, No. 1, Nan-Tai Street, Yungkang District, Tainan 71005, Taiwan

**Keywords:** virtual reality, musculoskeletal disorders, randomized controlled tria

## Abstract

To assess the effects of virtual reality on patients with musculoskeletal disorders by means of a scoping review of randomized controlled trials (RCTs). The databases included PubMed, IEEE, and the MEDLINE database. Articles involving RCTs with higher than five points on the Physiotherapy Evidence Database (PEDro) scale were reviewed for suitability and inclusion. The methodological quality of the included RCT was evaluated using the PEDro scale. The three reviewers extracted relevant information from the included studies. Fourteen RCT articles were included. When compared with simple usual care or other forms of treatment, there was significant pain relief, increased functional capacity, reduced symptoms of the disorder, and increased joint angles for the virtual reality treatment of chronic musculoskeletal disorders. Furthermore, burn patients with acute pain were able to experience a significant therapeutic effect on pain relief. However, virtual reality treatment of patients with non-chronic pain such as total knee replacement, ankle sprains, as well as those who went through very short virtual reality treatments, did not show a significant difference in parameters, as compared with simple usual care and other forms of treatment. Current evidence supports VR treatment as having a significant effect on pain relief, increased joint mobility, or motor function of patients with chronic musculoskeletal disorders. VR seems quite effective in relieving the pain of patients with acute burns as well.

## 1. Introduction

Virtual reality (VR) of players using body movement to interact with a computer is a new form of treatment in rehabilitation settings. It generates a virtual world in three-dimensional space through a computer simulation that stimulates user senses, such as sight and hearing, making users feel as if they are immersed in it. VR has three elements: Interaction, Immersion, and Imagination [1]. It can be used in the teaching of human anatomy, online navigation of museums, 3D game teaching, flight training, and rehabilitation [2]. VR has become a therapeutic tool in many medical and rehabilitation fields. Due to the cost decline and ease of use of this technology, it has become an effective tool and trend in various fields.

However, its greatest obstacles lie in the lack of space, time, support staff, appropriate customer and customer incentives, therapist knowledge, and management support. The clinical use of VR often depends on the motivation and attitude of the therapist [3,4].

In the clinical investigations on the VR experience and perception of physical therapists (PTs) and occupational therapists (OTs) in Canada conducted by Levac et al., it was found that VR treatment is most commonly used for stroke (25.8%), brain injury (15.3%), musculoskeletal disorder (14.9%), cerebral palsy (10.5%), and neurodevelopmental disorders (6.3%) [3]. Most of the clinical applications of VR are for neurological problems. Moreover, numerous literature shows that VR is used to treat patients with stroke, cerebral palsy, Parkinson’s disease, etc. [5,6,7,8,9,10]. Most researches in VR medical applications are used to the upper limb movement rehabilitation for stroke patients. The upper limb virtual reality rehabilitation systems were developed for the stroke group. The patient grasped and released the characteristic objects in the virtual environment, and finger movement control of the stroke patients after 4–6 weeks of VR intervention was improved [6,10]. Some scholars used Kinect and customized games to train the children with cerebral palsy (CP). The evidence appears to support the use of VR as a promising tool to be incorporated into the rehabilitation process of CP [7,11,12].

According to the World Heath Organization (WHO), musculoskeletal conditions affect muscles, bones, joints and associated tissues such as tendons and ligaments. To patients, musculoskeletal conditions are typically characterized by pain and limitations in mobility or functional ability.... Pain and restricted mobility are the consistent features of the range of musculoskeletal conditions. Musculoskeletal conditions are the second largest contributor to disability worldwide [13]. However, at present, there is less evidence on the therapeutic effect of VR on patients with musculoskeletal system disorder [14,15,16,17,18]. In addition, studies have shown that VR is beneficial in pain management, for example, in pain relief during dressing changes of burn patients [19]. VR can also reduce anxiety, distract from the fear of pain, and alleviate stress [20]. It can divert the attention of patients who are afraid of moving because of pain.

So far, there are no integrated and first-rate studies that explore which musculoskeletal disorders are suitable for VR treatment. The comparison of the effects of VR games and other treatments (e.g., traditional treatment, instrumental therapy, exercise) on patients with musculoskeletal disorder is inconclusive. Therefore, this article integrates the results of studies made in recent years into a scoping review to: (1) Compare the effectiveness of VR and other treatment interventions for patients with musculoskeletal disorder; (2) further explore whether there is any consistency in the VR treatment of patients with musculoskeletal system disorder, so as to give recommendations based on the highest level of evidence. This review only contains RCT articles with a PEDro Scale score ≥5 points.

## 2. Materials and Methods

### 2.1. Determination and Selection of Articles

The methodology of this scoping review was based on the Preferred Reporting Items for Systematic Reviews and Meta-Analyses (PRISMA) guidelines because the main aim of this work is mapping all the available literature in the musculoskeletal field [21]. The use of the checklists based on PRISMA statement improve the quality and transparency of the scoping reviews [17]. Search was made in the PubMed, IEEE, and the MEDLINE library for reference literature using keywords and synonyms of “virtual reality”, “pain”, and “musculoskeletal”. After performing a journal search, RCT (randomized controlled trial) journals that were written in English within the last 10 years (January 2008 to August 2018) were selected, and non-musculoskeletal diseases such as “stroke”, “neurological”, and “cognitive” were excluded using the Physiotherapy Evidence Database (PEDro) scale (http://www.pedro.org.au/). When reference materials could not be found on the PEDro website, scores were independently made by two authors who have completed the PEDro Scale training tutorial on the Physiotherapy Evidence Database.

**Figure 1:**
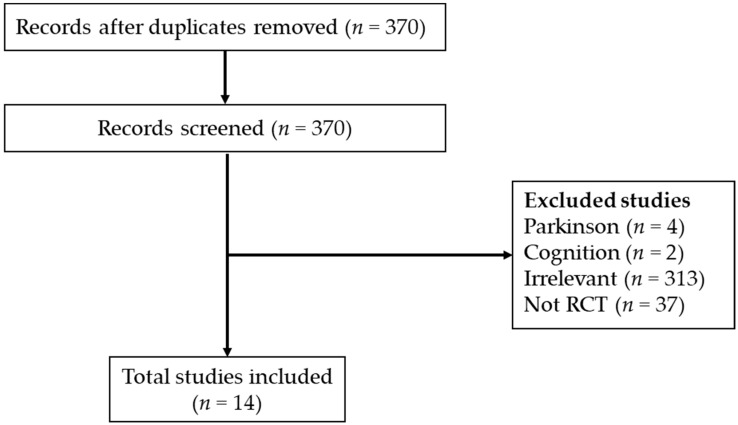
Flow chart displaying the screening process for studies included in this systematic review.

When the scores were different, the clinical physiotherapist with more than five years of experience, and who completed the PEDro assessment training, was asked to conduct another assessment. When issues such as disagreement or ambiguity arose, they were resolved through discussions. Finally, literature with very low PEDro scores (<5/10) was excluded. The search process is shown in Figure 1. Since there are few studies on VR for musculoskeletal disorders, we do not explore the virtual reality (VR) outcomes for any specific pathology in our study, but explore the VR treatment effects, such as pain relief, joint mobility, function, range of motion (ROM), muscle strength, angular velocity and self-satisfaction for all musculoskeletal disorders.

### 2.2. Data Extraction and Quality Assessment

Initially, the two authors completed the abstract review independently. When it was not possible to know whether an article could be included in the scoping review from its abstract, an assessment of the full article was made. All of the articles that had been included were reviewed in full. After sorting, the following were investigated: (1) Whether VR treatment improved the musculoskeletal system as compared with other treatments; (2) whether there was any consistency in the musculoskeletal disorder of patients that received VR treatment. The selected articles were summarized and analyzed with descriptive statistics. The author, publication year, subject, intervention, outcome measures, and mean between-group differences (95% confidence interval) were extracted from the references by the two authors of this study. A consensus was reached through discussion when the authors had different opinions.

## 3. Result

A database search was made to exclude articles with a PEDro score of less than 5 and non-English publications. A total of 14 articles were included. These 14 articles were included in this scoping review (Figure 1).

### 3.1. Quality of the Included Studies

The quality of included studies was presented in Table 1. The mean PEDro score of the included articles was 6.14 (range, 5–7). All studies were randomized (100%). 8 studies carry out concealed allocation (57.14%), and all studies baseline comparability (100%). All studies were analyzed between-group comparison (100%) and 13 studies reported point estimates and variability (92.86%). All studies didn’t carry out blind therapist. One study carried out blind subjects (7.14%) and 7 studies carried out blind assessors (50%). 10 studies have adequate outcome measurement (71.43%), and 5 studies have an intension-to-treat analysis (35.71%).

### 3.2. Description of Included Studies

Each article abstract (including author, musculoskeletal disorder, design, participants, intervention, comparison, and outcome measure) is organized in Table 2. In terms of age, a study about frozen shoulder investigated subjects older than 20 years of age [15]; a research about subacromial impingement syndrome (SAIS) studied subjects between 18–60 years old [22]; subjects of two articles discussing chronic cervical pain were older than 18 years old [17,18]; three studies that explored low back pain (LBP) had subjects between 18–50 years old [23], and those between 40–55 years old [24,25]; an investigation on pelvic floor muscle had subjects older than 50 years of age [26]; two researches discussed the treatment of acute burn wounds in adolescents aged 10–18 years [27,28]; three studies discussed the treatment for patients with TKR aged in the sixties [14,16,29]; an article discussing ankle sprains had subjects aged 18–64, belonging to the working-age group [30]. In terms of experimental intervention, most of the study regarding VR intervention lasted 15 to 30 min, 2 to 4 times per week for 2 to 6 weeks. One research conducted VR intervention for 3 weeks [16]; 3 articles discussed 4 weeks of VR intervention [15,17,24]; 2 studies described 5 weeks of VR intervention [18,26]; and another 2 articles discussed 6 weeks of VR intervention [22,30]. One study conducted VR treatment beginning the second day after TKA until 6 months [29]. There were 5 studies that compared VR intervention and no intervention at all [17,23,27,28,30]. The rest made comparisons between VR and other treatments.

### 3.3. Virtual Reality Resources Choosing

Virtual Reality was applied using several resources. In the 14 studies, one study used Kinect [15]; 5 studies used Wii [14,22,24,26,30]; 5 studies used VR glasses (one of the studies used headphones and joysticks) [17,18,25,27,28]; two studies used 3-D TV and 3-D shutter glasses [23,29]; and one study used enhanced reality with VR and mirror therapy [16].

### 3.4. Heterogeneity of Included RCT

The outcome could not be pooled into meta-analysis due to the following reasons. Clinical heterogeneity (Table 2) can be clearly observed from the participant, intervention, exercise mode, and outcome measures of the included studies. Diversity is seen in patient conditions, frequency and duration of VR intervention, whether or not the patient does home exercise, received patient health education, whether the experiment conducted was pure VR (only VR) or VR mixed with traditional physical therapy or with exercise therapy, whether the outcome measure contains follow-up, and whether different estimate measures were inconsistent at different times.

### 3.5. Effect of Virtual Reality versus Other Interventions

In the articles included, a total of twelve studies compared the effects of VR treatment and other intervention on orthopedic conditions (Table 3). The research on patients suffering from frozen shoulder for more than three months shows that four weeks of VR plus modalities (hot pack and ultrasound) produced a significant 8% increase in their shoulder range of motion (ROM) when compared to traditional exercise training, plus modalities [15]. Another research showed that after 6 months of short-term training and one-month of follow-up, the subacromial impingement syndrome (SAIS) patients without a rotator cuff problem on the VR group and home exercise group (scapular muscles training), were able to significantly reduce their disability and improve their quality of life. Furthermore, the VR group showed significant improvement of SAIS and scapular dyskinesis symptoms when compared with the home exercise group [22]. Another article showed that patients with chronic cervical pain who went through 5 weeks of VR and cervical kinematic training (KT) had a big difference in the global perceived change (variations in different areas of patient self-assessment, such as satisfaction, self-reported pain differences), which could last for three months when compared to those in the KT group [18]. A study also showed that after four weeks of training, the VR group of patients with chronic cervical pain displayed a significant difference in terms of pain, physical condition, fear of moving the neck, as well as in the mean and peak velocity from those in the laser beam projected group. However, there is no significant difference in cervical ROM during follow-up between the VR treatment group and the laser beam projected group [17]. Patients with chronic low back pain in another study were able to significantly improve pain, pressure algometry, disability, and the fear of low back pain after four weeks of VR training [24]. Another research proposed that VR with the supplementary traditional physical therapy can significantly reduce pain, fear, and increase functions for patients with subacute or chronic non-specific lower back pain [25]. Although a study comparing five weeks of pelvic floor muscle training via VR and traditional gym ball training, showed no significant difference in muscle strength, but a statistically significant difference in endurance was observed [26]. One study supported the idea that VR therapy during burn wound care can reduce adolescent pain [25]. Three included studies examined the effects of VR on patients with TKR [14,29]. The results of these two articles showed that VR treatment (physical therapy plus VR) did not produce a significant difference in terms of pain, ROM, walking speed, balance, and walking test for patients with total knee replacement (TKR), when compared with conventional therapy [14,16]. The other demonstrated that VAS scales were significantly lower in the experimental group than the control group during acute phase (at 3, 5, and 7 days after TKR) (*p* < 0.05) [29]. However, it did not reach the minimal clinically important difference (MCID) [31]. In the previously described study, VR intervention (one month, three months, six months after TKR) in the chronic phase can improve the functional recovery of the patients with TKR [29]. A study on the treatment of ankle sprains suggested that there is no significant difference in all parameters between VR treatment and traditional treatment [30].

### 3.6. Effect of Virtual Reality Versus No Intervention 

In the included articles, four articles discussed the therapeutic effects of VR and no intervention on chronic cervical pain, burn wound, low back pain, and ankle sprains (Table 4). When applied to chronic cervical pain and burn wound, a statistically significant difference was present in some parameters, as described in the following section. Bohat et al. (2017) [17] studied patients with chronic cervical pain after four weeks of training and found that the VR group had significantly different results from the control group in disability, cervical angular velocity, time to peak velocity, and head follow-up task accuracy. However, in cervical ROM, physical health, and fear of moving the neck, no significant difference was observed [17]. Another study compared the results of a 3-day VR training of low-back pain patients with the results of the non-invasive group, and found no statistically significant difference in lumbar spine flexion ROM and pain improvement [23].

During the dressing application of patients with burn wounds, patients undergoing VR treatment received significantly lower doses of Entonox (analgesic) compared with those in the standard distraction group. However, there is no significant reduction in patient pain [28]. For patients with ankle sprains, no statistically significant difference was observed between the VR treatment and the control group [30].

### 3.7. Effect of Virtual Reality on Acute and Chronic Musculoskeletal Pain

Although associated pain is not itself part of the root disorder, managing the pain of musculoskeletal disorders is a major part of general practice. Of the 14 musculoskeletal studies included, six were for acute pain, including the dressing of the burn wound [27,28], three were for TKR patients [14,16,29], one for patients with ankle sprain [30], and the rest of the eight articles were for chronic musculoskeletal pain patients, including patients with frozen shoulder, SAIS, Neck pain, LBP, and pelvic floor muscle training [15,17,18,22,24,25,26]. VR treatment seems to reduce the pain of burn patients, or it could reduce the use of analgesics [27,28]. No significant difference in all parameters was observed when TKR and ankle sprain patients received VR treatment as compared to conventional treatment [14,16,29,30]. And there is no MCID for VAS pain in its acute phase [29]. In the included articles, a significant difference in the main outcome was observed for all patients with chronic pain aside from the research conducted by Tomas et al. [23].

## 4. Discussion

Most virtual reality treatment research applications still focus on the VR treatment of central nervous system problems, such as stroke and cerebral palsy, while only a little research explores the therapeutic effect of VR treatment on patients with musculoskeletal disorders. At present, there is no research on the integration of virtual reality for patients with various musculoskeletal disorders and an analysis of its effects. Therefore, this scoping review searched and integrated multiple musculoskeletal disorders in VR applications. Analysis showed which patients with musculoskeletal disorders had better results after VR treatment.

In general, chronic pain usually lasts for more than 12 weeks, while acute pain usually lasts for 4 to 6 weeks [32]. Therefore, patients in the articles included in this study are those who experience chronic pain due to frozen shoulder (symptoms lasting more than 3 months), SAIS (symptoms lasting at least 2 months), neck pain (symptoms appear for more than 3 months), and LBP (symptoms persist for 2 or 3 months) [15,17,18,22,23,24,25]. The study on burn wound care included patients with acute pain due to burns. Fung et al. (2012) included TKR patients in their study under the condition of being able to apply a full load on the lower limbs after 2 weeks of physical therapy post-surgery. Another research made by Koo et al. studied TKR patients after 2 weeks of physical therapy post-surgery followed by VR treatment. In the preceding two TKR studies, patients belonged to the sub-acute and acute phase, and the pain that they felt was an acute pain [14,16]. As for another study, VR intervention was applied from one days to 6 months after TKR (longitudinal study). In the early postoperative period (3–7 days), VR intervention did not achieve any clinically better analgesic effect than traditional treatment [29]. The study on ankle sprains mentioned that patients with non-repetitive sprains can undergo emergency treatment for 4 weeks without pain followed by VR treatment; therefore, this does not belong to the category of chronic pain. From this systematic review, it was found that subjects that experienced more effective VR intervention tend to be patients with chronic orthopedic pain, or those with acute pain due to burn wounds. Patients with TKR and ankle sprains are not chronic pain patients, and results show that VR treatment is not more effective than other treatments.

Generally, patients suffering from chronic pain have lower levels of fitness than healthy people. This is because pain can affect the motor control strategies of people. Individuals tend to move in the least painful way; however, the least painful way is usually to refrain from moving. This causes a decrease in muscle size and strength; it repeatedly increases pain and stress, eventually producing to a vicious circle [33]. In the included research articles, the motions designed for patients with chronic orthopedic disorders are suitable for the joint movements of patients of this type. For example, in the virtual reality games for patients with frozen shoulder, the actions designed include shoulder elevation, shoulder IR/ER, and a shoulder abduction action, and suitable WII games are selected for shoulder impingement patients (such as the tennis game which involves shoulder capsule stretch, pectoral muscle stretch and shoulder elevation). For patients with other chronic orthopedic disorders, through somatosensory interactive games with larger movements, patients could try actions which they could not achieve. Furthermore, people usually focus on pain or impending pain; therefore, the use of VR is effective in distracting the attention of patients from pain. The distraction produced by VR reduces pain, induces movement, and promotes exercise. It also motivates patients to move. Most users describe that their experience of VR was pleasant, and it can relieve pain as well as reduce anxiety [20,34,35,36]. Nevertheless, VR treatment done under inadequate supervision may result in less than expected results [17,30]. VR intervention under supervision can increase the motivation or induce patients to receive movement training and boost their concentration. This systematic review found that VR treatment for patients with chronic pain, such as 4 weeks of VR plus modalities (hot pack and ultrasound) on patients with frozen shoulders produced a significant 8% increase in shoulder ROM when compared to traditional exercise training plus modalities [15]. Subacromial impingement syndrome (SAIS) patients underwent 6 weeks of VR training for 45 min/day, twice a week, and showed a significant improvement of SAIS and scapular dyskinesis symptoms than those in the home exercise group.

In addition, this result lasted for one month [22]. Another article that studied chronic cervical pain patients after 5 weeks of VR plus cervical kinematic training (KT) recounted a significant difference in global perceived change (patient self-reported changes in different areas, such as satisfaction, self-reported pain differences) when compared to the only KT group. The experienced outcome lasted for 3 months [18]. One research on low back pain showed that 4 weeks of VR training can alleviate pain, deep tissue pressure algometry, disability, and fear of low back pain. For the TKR patients, after one month to six months of VR intervention, the knee function is better than for those who received the traditional treatment [29]. One of the articles included in this study showed no significant difference in pain and lumbar spine flexion ROM after comparing 3 days of VR treatment for patients with lower back pain, and patients without VR treatment [23]. This scoping review shows that chronic patients may receive at least four weeks of VR treatment in order to experience a significant therapeutic effect. In addition, VR training seems to have a short-term effect for patients with chronic pain in the musculoskeletal system [17,18,22,25]. This is consistent with past research [36,37,38].

The results of this study show that VR treatment with a hand joystick significantly reduces the pain score of patients when removing dressings from patients with acute burns, or it will reduce the use of analgesics [27,28]. This is consistent with previous research [39,40,41,42]. Hoffman, et al. [40] showed that the use of VR for patients under severe pain can effectively reduce pain by 41%. It is speculated that VR can also be used to distract patients from severe acute pain during dressing change. Therefore, VR can be used to divert attention, thus reducing the use of analgesics.

In summary, VR treatment can reduce pain in acute burn wound care and chronic musculoskeletal disorders. It can effectively distract patients with chronic pain, and allow them to ignore the cumbersome rehabilitation training, consequently improving treatment motivation. In addition, VR treatment may be helpful in the psychological level and the establishment of confidence. For example, patients with burns or chronic disability may have a tendency to fall into depression because of the long course of the disorder. The use of VR can release psychological stress and reduce their fear of pain [19].

Virtual reality is also helpful in the control and perception of muscle movements. This systematic review includes the hard to control PFM, as well as waist and neck movements. The PFM training research included in this article [26] recommended the simple contraction of the lower abdominal muscles in the VR group. Previous studies pointed out that the lower abdominal muscles have a synergistic effect with PFM. Therefore, some scholars have suggested that if the patient does not know how to apply force during PFM contraction, training on abdominal transverse muscle contraction can be done to attain the same purpose [43]. Patients in the VR group interacted with the game screen and performed pelvic movements such as pelvic forward, backward, lateral tilt, and go around motions according to the easy-to-understand motion instructions provided by the Wii game screen. This made it possible for patients to understand how to control their pelvic motion, and at the same time, increased the control and perception of the PFM. The LBP patients included in this article used games that combined Wii and yoga for their training [24]. LBP patients usually have weak deep core muscles [44]. Yoga promotes the strengthening and relaxation of the waist muscles and ligaments. Through yoga, the body can be continuously aligned correctly. At the same time, the patients can clearly see their posture on the screen. The Wii board senses the weight and center of gravity of the body and trains LBP patients according to the steps in the game screen. For rehabilitation that needs repeated feedback and learning of exercises, VR can provide enthusiasm. Patients with neck pain can also use VR glasses to perform target tracking according to the instructions given by the game, and flex, stretch, and rotate the neck. Patients can adjust neck motion through the instant feedback given by the VR glasses [17,18]. The preceding discussions show that VR can be used to increase the control and training of PFM as well as the consciousness of waist and neck motion. Moreover, posture can be adjusted through VR instant feedback.

Depending on the different facilities which possess different visual perception methods, Virtual Reality can be divided into four types: (a) Desktop VR: Mouse, trackball, and joystick are the main computer transmission devices and a common PC screen was used as its output; (b) Simulator VR: In a specific environment, machines and equipment, added to an image screen, provided the Users simulation results; (c) Projection VR: With a large projection screen, several projectors, and stereo sound output devices, simulation scenes were projected around the user; (d) Immersion VR: Specific Input and output devices, such as helmet display, etc., were used in this type of simulation [1]. The result of the five included articles in the current study seemed to show some effectiveness of the immersion VR. VR glasses or VR TV output, 3D shuttle glasses or helmet display were used to allow the user to become fully immersed in the system, and computers were used to provide image or sound feedbacks (five out of five); Three of the five articles showed Wii (belong to the VR type (a) described as above ) had achieved some effectiveness. Some patients may have nausea and dizziness due to the problems of the VR device, such as mismatched motion, motion parallax, viewing angle, limited reproduction of a real environment, and the imperfect simulation of human–world interactions. This condition occurring may affect its treatment effectiveness [45]. Facing the current economic development and the increase of the need of clinical care, we believe that it is necessary to explore the clinical effectiveness and applicability of the VR system. This highlights the importance of the ongoing discussions of the MCID on pain relief or on function increase in this article. The challenges in using the truly immersive VR system include nausea or dizziness caused by immersing in the virtual world and investment costs (facilities, cost, personnel training) [35,45]. All of these also affect whether VR treatment is appropriate in clinical environment implementation.

### Limitations

Because this system review includes first-rate RCT studies, fewer articles that compare the effects of VR therapy with other interventions on patients with musculoskeletal disorders are available. In some articles, the lack of raw numeric data makes it impossible to calculate the mean difference between the experimental and control groups. During the article retrieval process, language was also restricted; therefore, some language bias might exist. In addition, very few articles contain the minimal clinically important difference (MCID) on various parameters; hence, further discussion was not made.

## 5. Conclusions

VR treatment appears to have a significant effect upon pain relief, increased joint mobility, or the motor functions of patients with chronic painful musculoskeletal disorders. VR seems quite effective in relieving the pain of patients with acute burns as well. However, there is insufficient evidence in the current literature; hence, more research is needed to explore the therapeutic effects of VR treatment on musculoskeletal disorders. In the future, VR games maybe used for more patients with chronic musculoskeletal injuries. As to whether different types of VR would affect the effectiveness for rehabilitation results in musculoskeletal disorder patients, this should also be further investigated.

## Figures and Tables

**Table 1:** Physiotherapy Evidence Database (PEDro) Score for Included Studies (*n* = 14).

	Huang et al. 2014 [15]	Pekyavas et al. 2017 [22]	Bahat et al. 2015 [18]	Bahat et al. 2017 [17]	Kim et al. 2014 [24]	Thomas et al. 2016 [23]	Yilmaz Yelvar et al. 2017 [25]	Martinho et al. 2016 [26]	Kipping et al. 2012 [28]	Jeffs et al. 2014 [27]	Fung et al. 2012 [14]	Koo et al. 2018 [16]	Jin et al. 2018 [29]	Punt et al. 2016 [30]
	2014	2017	2015	2017	2014	2016	2017	2016	2012	2014	2012	2018	2018	2016
	Taiwan	Turkey	Australia	Australia	Korea	America	Turkey	Brazil	Australia	America	Canada	Korea	China	Switzerland
Eligibility criteria	Y	N	Y	N	N	Y	Y	Y	Y	Y	Y	Y	Y	Y
Random allocation	Y	Y	Y	Y	Y	Y	Y	Y	Y	Y	Y	Y	Y	Y
Concealed allocation	N	N	Y	Y	N	Y	N	Y	Y	Y	N	Y	N	Y
Baseline comparability	Y	Y	Y	Y	Y	Y	Y	Y	Y	Y	Y	Y	Y	Y
Blind subjects	N	N	N	N	N	N	N	N	N	Y	N	N	N	N
Blind therapists	N	N	N	N	N	N	N	N	N	N	N	N	N	N
Blind assessors	Y	N	Y	Y	N	N	Y	N	N	N	Y	Y	N	Y
Adequate follow-up	Y	Y	Y	N	Y	Y	Y	N	Y	Y	Y	N	Y	N
Intention-to-treat analysis	Y	N	N	Y	N	N	N	Y	Y	N	N	N	N	Y
Between-group comparisons	Y	Y	Y	Y	Y	Y	Y	Y	Y	Y	Y	Y	Y	Y
Point estimates and variability	Y	Y	Y	Y	Y	Y	Y	Y	Y	Y	N	Y	Y	Y
Total score (0~10)	7/10	5/10	7/10	7/10	5/10	6/10	6/10	6/10	7/10	7/10	5/10	6/10	5/10	7/10

Abbreviations: PEDro, Physiotherapy Evidence Database; Y: yes; N: no.

**Table 2:** Description of Included studies.

Title	Author	Part	Design	Participant (number)		Intervention	Exercise Mode (Frequency or Intensity)	Outcome Measures
				Age (years) = mean (SD)				
Intelligent Frozen Shoulder Rehabilitation	Huang et al. 2014 [15]	Frozen shoulder	RCT	frozen shoulder syndrome > 3 months			20 min/time, 2 times/week (Total 4 weeks)	ROM, CMS assessment
				E	*n* = 20 Age (years) = 60.65 (11.84)	Hot pack + ultrasonic + VR		
				C	*n* = 20 Age (years) = 61.45 (12.84)	Hot pack + ultrasonic + traditional exercise training		
Comparison of virtual reality exergaming and home exercise programs in patients with subacromial impingement syndrome and scapular dyskinesis: Short term effect	Pekyavas et al. 2017 [22]	subacromial impingement syndrome(SAIS) & scapular dyskinesis	RCT	18–60 years old; Type II SAIS				VAS(rest, activity, night), SPADI, Neer, Hawkins, SRT, SAT, LSST1-3
				None rotator cuff problem				
				E	*n* = 15 Age (years) = 40.33 (13.20)	VR + control period (after 6 weeks)	VR: 45 min/day, twice a week, for 6 weeks; Control: 1 month for home exercise	
				C	*n* = 15 Age (years) = 40.60 (11.77)	Exercise + control period (after 6 weeks)	Exercise: 45 min/day, twice a week, for 6 weeks; Control: 1 month for home exercise	
Cervical Kinematic Training with and without Interactive VR Training for Chronic Neck Pain—a Randomized Clinical Trial	Bahat et al. 2015 [18]	Chronic neck pain	RCT	Neck pain > 3 months, NDI > 10%				VAS, Neck Disability Index, TSK, ROM, Peak velocity, mean velocity, TIP%, Sway SD, Accuracy, Eyes closed balance, singer leg stance, step test
				E	*n* = 16 Age (years) = 40.63 (14.18)	VR + kinematic training	Total 30 min, at least 3 times a week, for 5 weeks	
				C	*n* = 16 Age (years) = 41.13 (12.59)	kinematic training (using laser point)		
Remote kinematic training for patients with chronic neck pain: a randomized controlled trial	Bahat et al. 2017 [17]	Chronic neck pain	RCT	Neck pain > 3 months, NDI > 12%				Neck Disability Index, Peak velocity, mean velocity, VAS, EQ5D, TSK, NVP, TTP%, Accuracy, ROM, GPE
				VR	*n* = 30 Age (years) = 48 (9.5)	VR	1 set 5 min, 20 min/day, 4 times/week, for 4 weeks	
				Laser	*n* = 30 Age (years) = 48 (12.5)	Laser point training		
				C	*n* = 30 Age (years) = 48 (13)	Not receive any treatment		
The Effects of VR-Based Wii Fit Yoga on Physical Function in Middle-Aged Female LBP Patients	Kim et al. 2014 [24]	LBP	RCT	LBP > 2 months				VAS, pressure algometer, ODI, RMDQ, FBQ
				E	*n* = 15 Age (years) = 44.33	VR	30 min/session, 3 session/week, for 4 weeks (1 session had 7 exercise program. 3 min of exercise and 1 min of rest)	
				C	*n* = 15 Age (years) = 50.46	Trunk stabilizing exercise + physical therapy	2 sets (30 min), 1set included 10 repetitions, physical therapy 30 min	
Feasibility and Safety of a Virtual Reality Dodgeball Intervention for Chronic Low Back Pain: A Randomized Clinical Trial	Thomas et al. 2016 [23]	LBP	RCT	18–50 years old with LBP > 3 months				pain and harm, lumbar spine flexion ROM
				kinesiophobia ≥35				
				E	*n* = 26 Age (years) = 23.9 (6.8)	VR	3 days (<48 h)	
				C	*n* = 26 Age (years) = 26.7 (8.5)	Not receive any treatment		
Is physiotherapy integrated virtual walking effective on pain, function, and kinesiophobia in patients with non-specific low-back pain? Randomised controlled trial	Yilmaz Yelvar et al. 2017 [25]	LBP	RCT	non-specific LBP for longer than 2 months				VAS, ODI, TKS, TUG, and 6MWT scores
				E	*n* = 23 Age (years) = 46.27 (10.93)	VR + Traditional physical therapy	5 times/week, for 2 weeks	
				C	*n* = 23 Age (years) = 52.81 (11.53)	Traditional physical therapy	5 times/week, for 2 weeks	
The effects of training by virtual reality or gym ball on pelvic floor muscle strength in postmenopausal women: a randomized controlled trial	Martinho et al. 2016 [26]	Pelvic floor muscle	RCT	>50 years old women				Maximum strength, average strength, endurance
				>1 year postmenopausal phase				
				APT-VR	*n* = 30 Age (years) = 61.9 (8.6)	Abdominopelvic training by VR	1 session 5 min with 90 s resting, for 10 session. Twice a week, for 5 weeks	
				PFMT-GB	*n* = 30 Age (years) = 61 (8.5)	Pelvic floor muscle training using a gym ball	4 series of 10 fast & sustained (8 s maintain with 16 s resting), each exercise 5 times, twice a week, for 5 weeks	
Virtual reality for acute pain reduction in adolescents undergoing burn wound care: A prospective randomized controlled trial	Kipping et al. 2012 [28]	Burn wound	RCT	11–18 years old				VAS, FLACC scale
				burn wound Total Body Surface Area (TBSA) > 1%				
				E	*n* = 20 Age (years) = 12.6 (1.3)	VR	Dressing period (3–58 min), only 1 time	
				C	*n* = 21 Age (years) = 13.5 (1.8)	Another distraction way or no distraction		
Effect of Virtual Reality on Adolescent Pain During Burn Wound Care	Jeffs et al. 2014 [27]	Burn wound	RCT	10–17 years old				Adolescent Pediatric Pain Tool, Spielberger State-Trait Anxiety InventoryFor Children, Pre-Procedure Questionnaire, Post-Procedure Questionnaire
				standard care	*n* = 10 Age (years) = 18.9 (2.8)	standard care	Dressing period only 1 time	
				passive distraction	*n* = 10 Age (years) = 12.6 (2.1)	passive distraction watching a movie		
				virtual reality	*n* = 8 Age (years) = 14.8 (2.0)	virtual reality		
Use of Nintendo Wii Fit™ in the Rehabilitation of Outpatients Following Total Knee Replacement: a Preliminary Randomized Controlled Trial	Fung et al. 2012 [14]	TKR	RCT	requiring twice-weekly physiotherapy treatment for TKR rehabilitation				active knee flexion/extension ROM, 2 min walk test, NPRS, ABCS, LEFS
				Full lower extremity weight bearing				
				E	*n* = 27 Age (years) = 67.9 (9.5)	physiotherapy + VR	physiotherapy (45 min), 15 min VR until discharge	
				C	*n* = 23 Age (years) = 68.2 (12.8)	physiotherapy + lower extremity exercise	physiotherapy (45 min), 15 min lower extremity exercise until discharge	
Enhanced Reality Showing Long-Lasting Analgesia after Total Knee Arthroplasty: Prospective, Randomized Clinical Trial	Koo et al. 2018 [16]	TKR	RCT	Full term	*n* = 20 Age (years) = 63.7 (5.09)	VR + physiotherapy for 2 weeks	VR + PT: 5 days/week, for 2 weeks	VAS, WOMAC, 6 min walk test, Timed-stands test
				Half term	*n* = 22 Age (years) = 65.0 (6.97)	VR + physiotherapy for 1 week before physiotherapy for 1 week	VR + PT for 1 week	
Virtual reality intervention in postoperative rehabilitation after total knee arthroplasty: a prospective and randomized controlled clinical trial	Jin et al. 2018 [29]	TKR	RCT	E	*n* = 33 Age (years) = 66.45 (3.49)	VR(begin 2nd days for TKA) + conventional rehabilitation	three sets of 30 repetitions	WOMAC, HSS, VAS, ROM.
				C	*n* = 33 Age (years) = 66.30 (4.41)	conventional rehabilitation	three sets of 30 repetitions	
Wii Fit™ Exercise Therapy for the Rehabilitation of Ankle Sprains: Its Effect Compared with Physical Therapy or No Functional Exercises at All	Punt et al. 2016 [30]	Ankle sprain	RCT	18–64 years old				FAAM-ADL, FAAM-sport, VAS-rest, VAS-walk
				Grade I or II lateral ankle sprain				
				requiring 4 weeks RICE and can pain free movement				
				VR	*n* = 30 Age (years) = 34.7 (10.7)	VR	30 min/time, 2 times/week, for 6 weeks	
				Physiotherapy	*n* = 30 Age (years) = 34.7 (11.3)	modalities, joint mobilization, muscle strengthening, proprioceptive exercise	30 min/time, 9 times/6 weeks	
				C	*n* = 30 Age (years) = 33.5 (9.5)	Not receive any treatment	-	

Abbreviations: *n* (number); E (experimental group); C (control group); VR (Virtual reality); min (minute); ROM (range of motion); CMS (Constant-Murley score); VAS (Visual Analog Scale); SPADI (Shoulder Pain and Disability Index); SRT (Scapular Retraction Test); SAT (Scapular Assistance Test); LSST (Lateral Scapular Slide Test); TSK (Tampa scale of kinesiophobia); TIP% (Time to peak velocity percentage); GPE (Global perceived effect); sway SD (standard deviation of the static head sway); EQ-5D(EQ-5D™, http://www.euroqol.org); NVP (Number of velocity peaks); ODI (Oswestry low-back pain disability index); RMDQ (Roland Morris disability questionnaire); FBQ (fear avoidance beliefs questionnaire); NPRS (Numeric Pain Rating Scale); LEFS (Lower Extremity Functional Scale); ABCS (Activity-specific Balance Confidence Scale); WOMAC (Western Ontario and McMaster Universities Osteoarthritis Index); FAAM (Foot and Ankle Ability Measure); ADL (activities of daily living); RICE (rest, ice, compression and elevation); FLACC (Faces, legs, activity, cry, consolability scale); Hospital for Special Surgery knee score (HSS); TKS (TAMPA Kinesiophobia Scale), TUG (timed-up and go test); 6MWT (6-Minute Walk Test); RCT (randomized controlled trial); LBP (low back pain); TKR (Total Knee Replacement).

**Table 3:** Effect of virtual reality (VR) versus another intervention.

Study	Outcome Measure		Mean Difference between VR Groups and Another Intervention	Significance of Difference between Groups
Huang et al. 2014 [15]	ROM		8%	Between groups *p* < 0.05
	CMS		NA	Between groups *p* < 0.05
Pekyavas et al. 2017 [22]	Neer	post-intervention/1 month follow-up	NA	*p* = 0.02
	SRT	post-intervention/1 month follow-up	NA	*p* = 0.01
	SAT	post-intervention/1 month follow-up	NA	*p* = 0.047
	VAS (rest, activity, night)	post-intervention/1 month follow-up	NA	-
	SPADI	post-intervention/1 month follow-up	NA	-
	Hawkins	post-intervention/1 month follow-up	NA	-
	LSST1-3	post-intervention/1 month follow-up	NA	-
Bahat et al. 2015 [18]	cervical flexion ROM	post-intervention	NA	Between groups *p* < 0.05
		3 months follow-up	NA	-
	Global Perceived change	post-intervention	NA	-
		3 months follow-up	NA	Between groups *p* < 0.05
	VAS	post-intervention/3 months follow-up	NA	-
	NDI	post-intervention/3 months follow-up	NA	-
	TSK	post-intervention/3 months follow-up	NA	-
	Velocity	post-intervention/3 months follow-up	NA	-
	TIP%	post-intervention/3 months follow-up	NA	-
	Accuracy	post-intervention/3 months follow-up	NA	-
	sway SD	post-intervention/3 months follow-up	NA	-
	Eyes closed balance	post-intervention/3 months follow-up	NA	-
	singer leg stance	post-intervention/3 months follow-up	NA	-
Bahat et al. 2017 [17]	V_mean_ (F,LR)	Post-pre intervention	NA	Between groups *p* < 0.05
	V_peak_ (LR)	Post-pre intervention	NA	Between groups *p* < 0.05
	V_mean_ (F,E,LR)	3 months follow up-pre intervention	NA	Between groups *p* < 0.05
	V_peak_ (E,LR)	3 months follow up-pre intervention	NA	Between groups *p* < 0.05
	VAS	Post-pre/3 months-pre	NA	Between groups *p* < 0.05
	EQ5D	Post-pre/3 months-pre	NA	Between groups *p* < 0.05
	Accuracy (F,RR,LR)	Post-pre intervention	NA	Between groups *p* < 0.05
	Accuracy (F)	3 months follow up-pre intervention	NA	Between groups *p* < 0.05
	TTP%	Post-pre/3 months-pre	NA	Between groups *p* < 0.05
	ROM	Post-pre/3 months-pre	NA	-
	NDI	Post-pre/3 months-pre	NA	-
	TSK	Post-pre/3 months-pre	NA	-
	NVP	Post-pre/3 months-pre	NA	-
	GPE	Post-pre/3 months-pre	NA	-
Kim et al. 2014 [24]	VAS		NA	Between groups *p* < 0.05
	Pressure algometer		NA	
	ODI		NA	
	FBQ		NA	
	RMDQ		NA	-
Yilmaz Yelvar et al. 2017 [25]	VAS		NA	Between groups *p* < 0.05
	TKS		NA	
	TUG		NA	
	6 MWT scores		NA	
Martinho et al. 2016 [26]	Maximum strength		−0.08	*p* = 0.1
	average strength		0.01	*p* = 0.6
	Endurance		1.83	*p* = 0.007
Jeffs et al. 2014 [27]	Pain		23.7	*p* = 0.029
Fung et al. 2012 [14]	Active knee flexion ROM		−0.33	-
	Active knee extension ROM		−0.6	-
	2 min walk test		2.68	-
	NPPS		16.84	-
	ABCS		14.11	-
	LEFS		31.85	-
Koo et al. 2018 [16]	VAS		NA	-
	WOMAC		NA	-
	6 min walk test		NA	-
	Timed-stands test		NA	-
Jin et al. 2018 [29]	VAS (at 3, 5, 7 days after TKR)		NA	*p* < 0.05
	WOMAC (at 1, 3, 6 months after TKR)		NA	*p* < 0.05
	HSS (at 1, 3, 6 months after TKR)		NA	*p* < 0.05
Punt et al. 2016 [30]	FAAM-ADL		NA	-
	FAAM-sport		NA	-
	VAS-rest		NA	-
	VAS-walk		NA	-

Abbreviations: VR (Virtual reality); ROM (range of motion); CMS (Constant-Murley score); VAS (Visual Analog Scale); SPADI (Shoulder Pain and Disability Index); SRT (Scapular Retraction Test); SAT (Scapular Assistance Test); LSST (Lateral Scapular Slide Test); NDI (Neck Disability Index); TSK (Tampa scale of kinesiophobia); TIP% (Time to peak velocity percentage); GPE (Global perceived effect); sway SD (standard deviation of the static head sway); EQ-5D(EQ-5D™, http://www.euroqol.org); NVP (Number of velocity peaks); ODI (Oswestry low-back pain disability index); RMDQ (Roland Morris disability questionnaire); FBQ (fear avoidance beliefs questionnaire); NPRS (Numeric Pain Rating Scale); LEFS (Lower Extremity Functional Scale); ABCS (Activity-specific Balance Confidence Scale); WOMAC (Western Ontario and McMaster Universities Osteoarthritis Index); FAAM (Foot and Ankle Ability Measure); ADL (activities of daily living); v_mean_ (Mean velocity); Vpeak (Peak velocity); F (Flexion), E (Extension), LR (Left rotation), RR (Right rotation); the-marked mean *p* > 0.05; NA (not available); Hospital for Special Surgery knee score (HSS); TKS (TAMPA Kinesiophobia Scale), TUG (timed-up and go test); 6 MWT (6-Minute Walk Test).

**Table 4:** Effect of VR versus no intervention.

Study	Outcome Measure	Mean Difference between VR Groups and Control Group	Significance of Difference between Groups
Bahat et al. 2017 [17]	NDI	NA	Between groups *p* < 0.05
	velocity	NA	
	TTP% (F,LR)	NA	
	Accuracy (F,RR)	NA	
	ROM	NA	-
	EQ5D	NA	-
	TSK	NA	-
	NVP	NA	-
Thomas et al. 2016 [23]	ROM	NA	-
	Pain	NA	-
Kipping et al. 2012 [28]	VAS	NA	-
	FLACC (dressing removal)	NA	Between groups *p* < 0.05
Punt et al. 2016 [30]	FAAM-ADL	NA	-
	FAAM-sport	NA	-
	VAS-rest	NA	-
	VAS-walk	NA	-

Abbreviations: VR (Virtual reality); NDI (Neck Disability Index); TIP% (Time to peak velocity percentage); ROM (range of motion); EQ-5D (EQ-5D™, http://www.euroqol.org); TSK (Tampa scale of kinesiophobia); NVP (Number of velocity peaks); VAS (Visual Analog Scale); FLACC (Faces, legs, activity, cry, consolability scale); FAAM (Foot and Ankle Ability Measure); ADL (activities of daily living); the-marked mean *p* > 0.05; NA (not available).
